# RNA binding protein AUF1/HNRNPD regulates nuclear export, stability and translation of *SNCA* transcripts

**DOI:** 10.1098/rsob.230158

**Published:** 2023-11-22

**Authors:** Fedon-Giasin Kattan, Pelagia Koukouraki, Athanasios K. Anagnostopoulos, George T. Tsangaris, Epaminondas Doxakis

**Affiliations:** ^1^ Center of Basic Research, Biomedical Research Foundation of the Academy of Athens (BRFAA), Soranou Efesiou 4, Athens 11527, Greece; ^2^ Department of Biological Applications and Technology, Faculty of Health Sciences, University of Ioannina, 45110 Ioannina, Greece

**Keywords:** SNCA, AUF1/HNRNPD, post-transcriptional regulation, nucleocytoplasmic shuttling, deadenylation, RNA binding proteins

## Abstract

Alpha-synuclein (SNCA) accumulation plays a central role in the pathogenesis of Parkinson's disease. Determining and interfering with the mechanisms that control SNCA expression is one approach to limiting disease progression. Currently, most of our understanding of SNCA regulation is protein-based. Post-transcriptional mechanisms directly regulating *SNCA* mRNA expression via its 3′ untranslated region (3′UTR) were investigated here. Mass spectrometry of proteins pulled down from murine brain lysates using a biotinylated *SNCA* 3′UTR revealed multiple RNA-binding proteins, of which HNRNPD/AUF1 was chosen for further analysis. AUF1 bound both proximal and distal regions of the *SNCA* 3′UTR, but not the 5′UTR or CDS. In the nucleus, AUF1 attenuated *SNCA* pre-mRNA maturation and was indispensable for the export of *SNCA* transcripts. AUF1 destabilized *SNCA* transcripts in the cytosol, primarily those with shorter 3′UTRs, independently of microRNAs by recruiting the CNOT1-CNOT7 deadenylase complex to trim the polyA tail. Furthermore, AUF1 inhibited *SNCA* mRNA binding to ribosomes. These data identify AUF1 as a multi-tasking protein regulating maturation, nucleocytoplasmic shuttling, stability and translation of *SNCA* transcripts.

## Graphical abstract

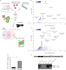

## 1. Introduction

Alpha-synuclein (SNCA) is an abundant presynaptic protein that functions as a SNARE-complex chaperone involved in the modulation of synaptic neurotransmission. Converging evidence has implicated SNCA in the pathogenesis of Parkinson's disease (PD) and some other diseases categorized as alpha-synucleinopathies. Several families with autosomal dominant early-onset PD have had point mutations, gene duplications, and gene triplications at the *SNCA* locus [1–3]. Viral-mediated overexpression of wild-type or mutant SNCA within nigral neurons of rodents and non-human primates resulted in progressive motor dysfunction resembling the motor symptoms of PD patients [4–6]. Moreover, SNCA is a major component of neuronal cytoplasmic deposits known as Lewy bodies (LB) in sporadic PD and dementia with Lewy bodies (DLB) and other proteinaceous inclusions in both glial and neuronal cells in multiple system atrophy (MSA) [7]. The exact causes of SNCA neurodegeneration are unknown, but misfolded and clumped forms are released from neurons, which help the pathology spread like a prion (reviewed by [8]). Taken together, these studies suggest that maintaining SNCA expression at physiological levels is an essential aspect of neuronal function and viability.

In eukaryotes, gene activity is not directly reflected by RNA levels because processing, transport, stability and translation are co- and post-transcriptionally regulated. RNA-binding proteins (RBPs) that bind to specific cis-elements in the promoters of genes and along the RNA sequence tightly control and run these processes. Due to the architectural complexity of neurons, exemplified by a relatively small soma and a vast network of projections and connections, the functional role of RBPs is vital within the nervous system. RBPs are essential for diverse facets of neurogenesis, neurite outgrowth, synapse formation, and plasticity (reviewed in [9,10]). Among the large family of RBPs, translation and turnover regulatory (TTR)-RBPs [11] modulate mRNA turnover and translation by interacting with U-rich elements in target mRNAs' 3′UTRs. The heterogeneous nuclear ribonucleoprotein D (HNRNPD), also known as AU-binding factor 1 (AUF1), is the first TTR-RBP that has been isolated and shown to control the stability of mRNAs [12]. AUF1 pre-mRNA alternative splicing generates four protein isoforms (p37, p40, p42 and p45) that shuttle between the nucleus and cytoplasm and may exhibit differential affinity for target transcripts [13]. AUF1 regulates multiple processes in the nucleus, such as telomere maintenance, transcriptional activation, and alternative splicing [13]. In the cytoplasm, it binds U-, GU- and UG-rich sequences and either promotes decay or enhances the stability and translation of target mRNAs by mechanisms that are still poorly understood [14]. AUF1 deficiency accelerates ageing in mice and increases senescence in both mouse and human cells [15].

The *SNCA* mRNA possesses a highly conserved and U-rich 3′UTR of about 575 nucleotides (nt) that is alternatively polyadenylated in humans to produce a transcript of 2.529 nt. The more extended transcript variant shows a similar expression to shorter transcripts and has been linked to PD pathology [16]. Further, the mRNAs of presynaptic genes, like *SNCA*, display significantly longer 3′UTRs than all other cellular transcripts, indicating that they are biased toward post-transcriptional regulation of expression [17]. Based on the above, we surveyed the 2.5 kb *SNCA* 3′UTR sequence for interacting RBPs and pulled AUF1 among several other RBPs. We found that AUF1 interacts stronger with the distal part of *SNCA* 3′UTR, attenuates *SNCA* pre-mRNA maturation, and is indispensable for *SNCA* mRNA nuclear export. Moreover, AUF1 destabilizes *SNCA* mRNA independently of microRNAs by recruiting CNOT1-CNOT7 deadenylase complex to shorten the polyA tail and blocks *SNCA* mRNA ribosomal engagement in the cytosol.

## 2. Results

### 2.1. Mass spectrometry proteomics unveils AUF1's association with *SNCA* mRNA in the brain

**Figure 1.**
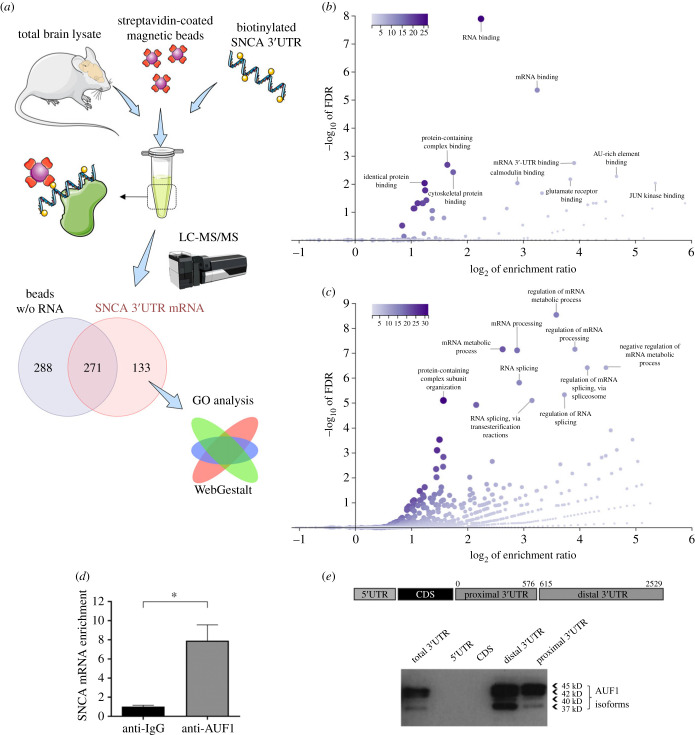
AUF1 associates with *SNCA* 3′UTR. (*a*) Schematic of the experimental procedure used to identify RBPs that bind the 2.5 kb *SNCA* 3′UTR. (*b*) GO ‘molecular function’ and (*c*) ‘biological process’ categories of proteins bound to *SNCA* 3′UTR. (*d*) RNA immunoprecipitation analysis was performed using non-specific IgG or anti-AUF1 antibody to confirm the interaction between endogenous AUF1 and *SNCA* mRNA. (*e*) Biotinylated *SNCA* RNA fragments were incubated with SK-N-SH cell lysates, and the presence of AUF1 in the pull-down material was assessed by Western blotting. AUF1 is bound exclusively to the SNCA 3′UTR in proximal and distal portions. Some elements in this image were obtained from Servier Medical Art (http://smart.servier.com/), permissible to use under a Creative Commons Attribution 3.0 Unported License.

To identify cellular proteins that specifically interact with the regulatory 3′UTR of *SNCA* mRNA, a biotinylated RNA spanning the 2529 nt *SNCA* 3′UTR was synthesized and incubated with total brain lysates from postnatal day three mouse brains. Then, the resulting RNP complexes were pulled down using streptavidin-coated magnetic beads, and nano LC-MS/MS analysis was conducted to identify all interacting proteins. Streptavidin beads non-specifically bound 559 proteins, while those with the biotinylated *SNCA* RNA attached pulled 404 proteins. After filtering out the latter dataset for non-specific labelling by deducting proteins bound to streptavidin beads alone, 133 unique proteins remained (figure 1*a*). Next, the pulled-down proteins' molecular functions and biological processes were analysed using the Gene Ontology (GO) database of the WebGestalt toolkit. *SNCA* 3′UTR-bound proteins displayed predominantly ‘RNA binding’ (E/R 4.8, FDR 1.3 × 10^−8^), ‘mRNA binding’ (E/R 9.5, FDR 4.5 × 10^−6^) and ‘mRNA 3′UTR binding’ (E/R 15, FDR 1.8 × 10^−3^) activities (figure 1*b*). The top three biological processes that were enriched were ‘regulation of mRNA metabolic process' (E/R 12, FDR 2.9 × 10^−9^), ‘mRNA metabolic process’ (E/R 6.2, FDR 7.1 × 10^−8^) and ‘regulation of mRNA processing’ (E/R 15.1, FDR 7.1 × 10^−8^) (figure 1*c*). These results validate the particular protocol for discovering 3′UTR interactors *en masse* and indicate that *SNCA* 3′UTR interacting proteins are implicated in mRNA metabolism.

Given that ‘AU-rich element binding’ was one of the top enriched molecular functions of the pulled proteins (figure 1*b*) and that *SNCA* 3′UTR has several long stretches of U nt that are likely to be implicated in mRNA stability and translation, AUF1 was chosen to investigate if it has a regulatory role in *SNCA* expression. Initially, to verify the proteomics finding, we assessed whether AUF1 associates with the *SNCA* mRNA by performing RNA immunoprecipitation (RIP) with anti-AUF1 antibody in human neuroblastoma SK-N-SH cells under native conditions (that is, without UV crosslinking). RNA isolated from IgG (control) and AUF1 RIPs was then subjected to real-time RT-PCR to monitor *SNCA* mRNA levels; *GAPDH* mRNA, ‘contaminating’ at low levels all RIP samples, served as a normalizing control. Figure 1*d* shows that *SNCA* mRNA was eight-fold enriched in AUF1 IP compared with control IgG IP, confirming that it is part of the AUF1 RNP complexes. We next explored whether AUF1 associates with *SNCA* mRNA domains other than the 3′UTR. Four biotinylated RNA transcripts spanning the 5′UTR, the CDS, the first 575 nt of *SNCA* 3′UTR, and the next 1900 nt of *SNCA* 3′UTR were prepared (figure 1*e* schematic view). These were then incubated with SK-N-SH lysate and streptavidin-coated beads to pull down RNP complexes. Western blotting revealed that AUF1 bound exclusively to the *SNCA* 3′UTR in both proximal and distal portions, with a greater number of AUF1 species binding the latter, indicating multiple interactions as predicted from the abundance of U-rich stretches throughout the sequence and competition with other RBPs for proximal binding (figure 1*e*).

### 2.2. AUF1 promotes *SNCA* mRNA decay

**Figure 2.**
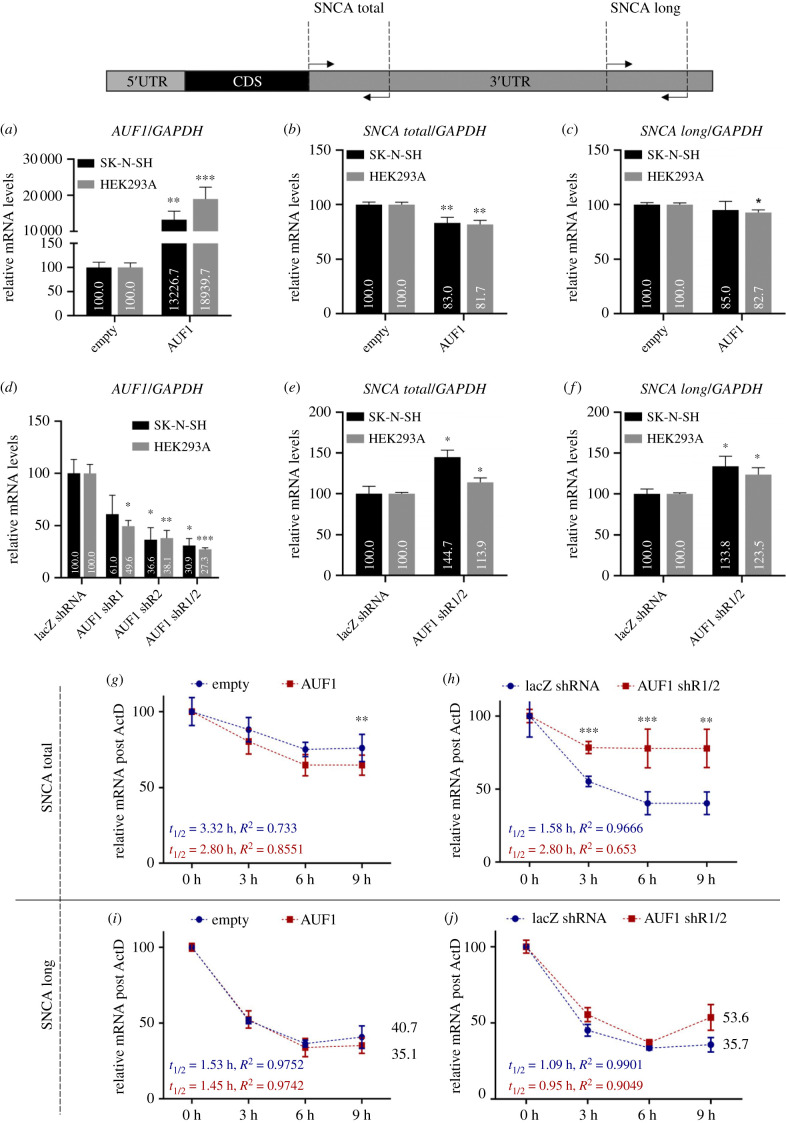
AUF1 destabilizes predominantly short *SNCA* transcripts to attenuate total *SNCA* mRNA expression. (*a–f*) Forty-eight hours after plasmid-mediated AUF1 overexpression (*a–c*) or silencing (*d–f*) in SK-N-SH and HEK293A cells, RNA was extracted, and steady-state levels of *SNCA* transcripts were assessed using RT-qPCR. AUF1 overexpression decreased total *SNCA* transcript levels, while AUF1 silencing increased total and long *SNCA* transcript levels in both cell lines. (*g–j*) The *SNCA* mRNA stability, 48 h after AUF1 overexpression (*g,h*) or silencing (*i,j*), was investigated by treating SK-N-SH cells with actinomycin D, extracting total RNA at 0, 3, 6, and 9 h, and measuring *SNCA* mRNA levels by RT-qPCR. AUF1 overexpression did not significantly decay *SNCA* mRNA, whereas AUF1 silencing significantly increased total *SNCA* mRNA stability but not the stability of the *SNCA* transcript with the long 3′UTR. Data show the mean ± s.d. from at least 3 biological replicates (*p < 0.05, **p < 0.01, ***p < 0.001).

Having confirmed the binding of AUF1 to the *SNCA* 3′UTR, we next examined the AUF1 functional role in *SNCA* mRNA expression by modulating its levels in human neuroblastoma SK-N-SH and embryonic adrenal precursor HEK293A cells, two cell lines that express high levels of endogenous *SNCA* mRNA and protein. Plasmid-mediated overexpression of AUF1 in SK-N-SH and HEK293A cells for 48 h, followed by RT-qPCR analysis of RNA extracts, reduced steady-state total levels of endogenous *SNCA* mRNA by approximately 18% (*p* < 0.01) in both cell lines. The expression of the *SNCA* transcript with the 2529 nucleotides 3′UTR (hereafter called ‘long transcript’) did not significantly change in SK-N-SH cells but decreased by 7% (*p* < 0.05) in HEK293A cells (figure 2*a–c*). Conversely, silencing AUF1 expression by overexpressing two different AUF1 shRNA plasmids for 48 h increased total *SNCA* transcript levels by 45% (*p* < 0.05) in SK-N-SH and 14% (*p* < 0.05) in HEK293A, while the long *SNCA* transcript levels increased by 34% (*p* < 0.05) in SK-N-SH cells and by 24% (*p* < 0.05) in HEK293A cells (figure 2*d–f*).

Given that modulating AUF1 levels changed *SNCA* mRNA abundance, we next investigated whether this effect was due to the differential regulation of mRNA stability. Forty-eight hours after transfection of SK-N-SH cells with AUF1 plasmids, cells were treated with actinomycin D to block *de novo* transcription, and the half-life of *SNCA* mRNA was measured by examining the rate of its clearance. Figure 2*g,h* show that AUF1 overexpression marginally decreased total *SNCA* mRNA half-life, while AUF1 silencing increased total *SNCA* mRNA expression by nearly 40% (*p* < 0.01). Following the steady-state expression data, AUF1 did not significantly affect the stability of the *SNCA* transcript with the long 3′UTR (figure 2*i,j*).

Overall, this set of experiments demonstrates that AUF1 destabilizes mostly the *SNCA* transcripts with the shorter 3′UTRs, resulting in significant downregulation of total *SNCA* mRNA levels. This effect is diminished on the long *SNCA* transcript, likely due to the interference from additional RBPs bound on the distal segment of *SNCA* 3′UTR.

### 2.3. AUF1 deadenylates the polyA tail of *SNCA* mRNA by recruiting the CNOT1-CNOT7 deadenylase complex

**Figure 3.**
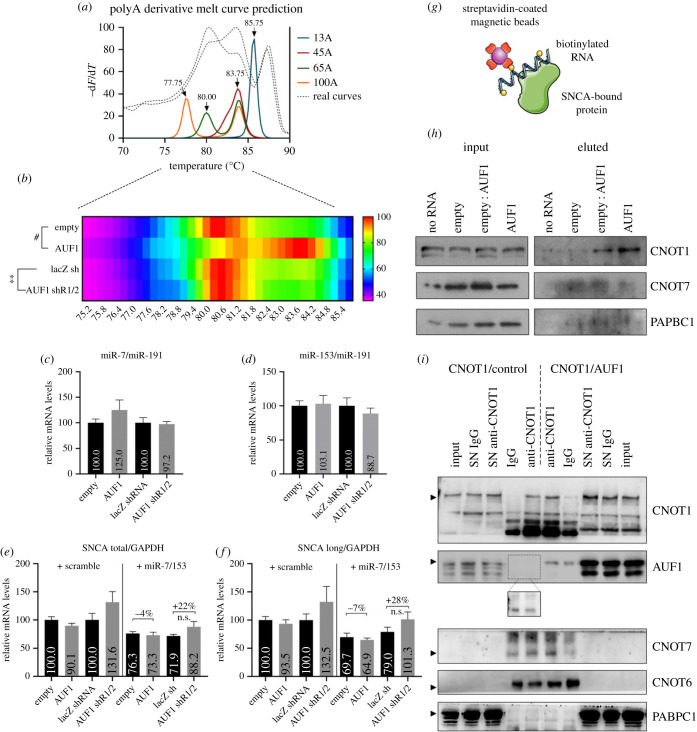
AUF1 recruits the CNOT1-CNOT7, but not the miRISC, complex to deadenylate *SNCA* transcripts. (*a*) Melting curve analysis of SNCA RT-qPCR products having 13, 45, 65 or 100 adenosine residues as predicted by uMeltQUARTZ. The shorter the polyA tail, the higher the melting temperature. (*b*) Acquired melting curves of RT-qPCR products following AUF1 overexpression or silencing in SK-N-SH cells after 48 h. AUF1 overexpression shifted PCR products toward higher Tm, while AUF1 silencing shifted PCR products toward lower Tm. (*c,d*) SK-N-SH cells were collected 48 h after AUF1 overexpression or silencing, and the levels of mature miR-7 and miR-153 were measured using RT-qPCR. AUF1 expression did not affect their expression. (*e,f*) SK-N-SH cells were collected 48 h after plasmids expressing scramble or both miR-7 and miR-153 in tandem were co-transfected with either AUF1 expressing or silencing plasmids, and the levels of *SNCA* transcripts were measured using RT-qPCR. AUF1 expression did not significantly affect the potency of miRNA inhibition on total or long *SNCA* transcript expression. (*g*) Schematic view of the assay used to pull down proteins associated with *SNCA* mRNA following AUF1 overexpression in SK-N-SH cells for 48 h. (*h*) Increased expression of AUF1 induces CNOT1, but not CNOT7 or PABPC1, binding to long *SNCA* 3′UTR. (*i*) To identify proteins that associate with CNOT1, immunoprecipitation with IgG (control) or anti-CNOT1 antibody was conducted in SK-N-SH cells 48 h after they were co-transfected with CNOT1 and either empty- or AUF1-expressing plasmids. CNOT1 immunoprecipitated AUF1, CNOT7 and PABPC1, but not CNOT6. SN: supernatant of the lysate after immunoprecipitation. Some elements in this image were obtained from Servier Medical Art (http://smart.servier.com/), permissible to use under a Creative Commons Attribution 3.0 Unported License.

Changes in the polyA tail length have long been recognized as a hallmark of the switch between mRNA stability and degradation. To investigate if AUF1 affects polyadenylation of *SNCA* mRNA, SK-N-SH cells transfected with the plasmids overexpressing or silencing AUF1 were analysed using a modified protocol of the Extension PolyA Test (ePAT) method [18]. Figure 3*a* shows the melting curve (Tm) of different (13, 45, 65, 100) polyadenosine (polyA) RT-qPCR products predicted by uMeltQUARTZ software using PCR primers specific to *SNCA* mRNA, while figure 3*b* displays the acquired melting curves following AUF1 overexpression or silencing in SK-N-SH cells for 48 h. AUF1 overexpression shifted PCR products toward higher Tm, indicative of shorter polyA, while AUF1 silencing shifted PCR products toward lower Tm, indicative of longer polyA. These findings suggest that AUF1 downregulates *SNCA* mRNA levels by destabilizing *SNCA* mRNA via deadenylation.

Next, the mechanism by which AUF1 drives *SNCA* mRNA deadenylation was examined. One hypothesis is that AUF1 either enhances the expression of neuronal miR-7 and miR-153, which regulate *SNCA* mRNA levels or promotes their recruitment on *SNCA* 3′UTR, as previously suggested for other miRNAs and AUF1 target mRNAs, respectively [19,20]. Accordingly, RT-qPCR analysis of miR-7 and miR-153 expression was conducted on RNA extracts from SK-N-SH cells transfected with the plasmids overexpressing or silencing AUF1. Figure 3*c,d* show that neither mature miR-7 nor miR-153 expression was affected by AUF1 levels. Next, a plasmid co-expressing miR-7 and miR-153 was co-transfected with the plasmids overexpressing or silencing AUF1 in SK-N-SH cells. Forty-eight hours later, RT-qPCR analysis of *SNCA* mRNA expression revealed that AUF1 overexpression did not affect miR-7/153-mediated *SNCA* mRNA degradation (figure 3*e,f*). AUF1 silencing decreased by 22% and 28% the effect of miR-7/153 on total and long *SNCA* mRNA levels, yet these results were statistically insignificant (*p* = 0.12 for total and *p* = 0.18 for long *SNCA* mRNA). These findings suggest that, in the absence of AUF1, miR-7/153 may not be as efficient in lowering *SNCA* levels, but AUF1 appears to be generally dispensable for their function on *SNCA* mRNA. The CCR4–NOT (carbon catabolite repression 4-negative on TATA-less) complex, the major deadenylase in mammals, is formed by CNOT1 that acts as a scaffold to about seven subunits of which CNOT6 and CNOT7/CAF1 are the catalytic members. To investigate if AUF1 recruits this complex on *SNCA* 3′UTR, the biotinylated *SNCA* 3′UTR was incubated with total lysates from SK-N-SH cells, 48 h after they were transfected with varied quantities of AUF1 expressing plasmid. Figure 3*h* shows that, by increasing the quantity of AUF1 available in lysates, there was an increased association of CNOT1 with *SNCA* 3′UTR. This association was unaccompanied by a catalytic CNOT7 pull-down on *SNCA* 3′UTR, presumably because the assay does not favour third-order interactions. To test if CNOT7 is pulled down with CNOT1, CNOT1 complexes were immunoprecipitated from total extracts from SK-N-SH cells co-transfected with CNOT1 and either empty- or AUF1-expressing plasmids. Figure 3*i* shows that CNOT1 is successfully immunoprecipitated with a CNOT1-specific antibody, yet there is some non-specific association of CNOT1 protein with the magnetic beads. Importantly, CNOT1 immunoprecipitated with AUF1 (1.6-fold enrichment), confirming the above pull-down data, and with CNOT7 (3-fold enrichment), validating previous studies [21,22]. However, CNOT1 showed no interaction with CNOT6, and there appeared to be binding to PABPC1, which is perhaps unexpected because it protects polyA sites from degradation. These findings indicate that AUF1 mediates the deadenylation of *SNCA* mRNA by directly recruiting the CNOT1-CNOT7 complex to *SNCA* 3′UTR and not through the miR7/153-GW182-CNOT1 pathway [23].

### 2.4. Discordant role of AUF1 on SNCA protein expression

**Figure 4.**
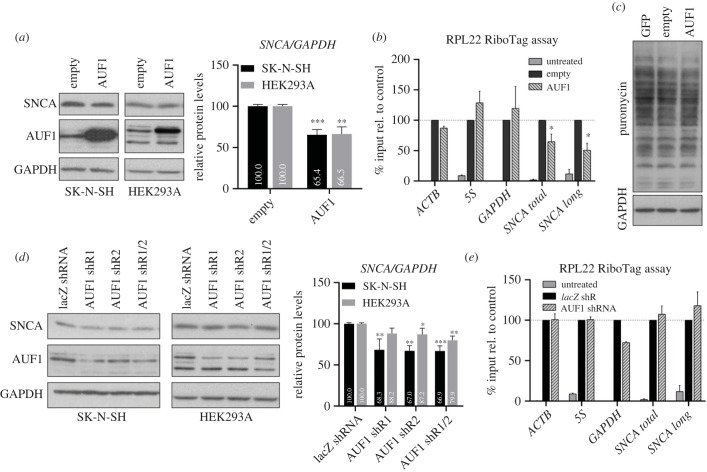
Deregulation of AUF1 levels lower SNCA protein expression. (*a*) Forty-eight hours after transfecting SK-N-SH and HEK293A cells with an AUF1 or empty plasmid, the protein levels of SNCA were analysed by Western blotting. AUF1 overexpression significantly decreased SNCA protein levels in both cell lines. (*b*) RiboTag analysis was performed in SK-N-SH cells co-transfected with plasmids expressing AUF1 and flagged ribosomal protein RPL22 to assess the interaction between *SNCA* transcripts and ribosomes. AUF1 overexpression inhibited *SNCA* transcript association with ribosomes. (*c*) Forty-eight hours after transfecting SK-N-SH cells with an empty or AUF1 plasmid, cells were treated with puromycin, and global protein synthesis rates were assessed by Western blotting using an anti-puromycin antibody. AUF1 overexpression did not affect global translation rates. (*d*) Forty-eight hours after transfecting SK-N-SH and HEK293A cells with AUF1 shRNA plasmids, the protein levels of SNCA were analysed by Western blotting. AUF1 silencing significantly decreased SNCA protein levels in both cell lines. (*e*) RNA immunoprecipitation analysis of SK-N-SH cells co-transfected with plasmids expressing AUF1 shRNA and flagged ribosomal protein RPL22 revealed that AUF1 silencing does not significantly affect *SNCA* mRNA association with ribosomes. Data show the mean ± s.d. from at least 3 biological replicates (*p < 0.05, **p < 0.01, ***p < 0.001).

Next, the effect of AUF1 on SNCA protein expression was determined. Western blotting of total protein extracts from AUF1 overexpressing SK-N-SH and HEK293 cells revealed a 35% (*p* < 0.001) and 33% (*p* < 0.01) decrease in total SNCA protein levels after 48 h, respectively (figure 4*a*). This outcome agrees with the decrease in steady-state mRNA levels observed following AUF1 overexpression. To investigate if it results from mRNA loss alone or if translation inhibition is also implicated, the ribosomal association of *SNCA* mRNA was tested in SK-N-SH cells expressing the flagged ribosomal protein RPL22 upon overexpressing or silencing AUF1 for 48 h. RPL22-IP with anti-flag beads followed by real-time RT-PCR to monitor *SNCA* mRNA levels revealed a 35% (*p* < 0.05) reduction in the association of total *SNCA* transcripts with the ribosomes following AUF1 overexpression. Similarly, the levels of the *SNCA* transcript with the long 3′UTR were reduced by 49% (*p* < 0.05). The association of three control mRNAs, *5S*, *ACTB* and *GAPDH*, with the ribosomes was unaffected by AUF1 overexpression, indicating that the effect of AUF1 was specific to *SNCA* mRNAs (figure 4*b*). In addition, measuring global protein synthesis rates using the non-isotopic SUnSET method [24] revealed that AUF1 overexpression does not generally affect translation in SK-N-SH cells, further reinforcing the specificity of this regulation (figure 4*c*). Overall, these findings suggest that excess AUF1 attenuates SNCA protein levels by two alternative mechanisms: (i) destabilizing shorter *SNCA* transcripts and (ii) blocking the translation of all *SNCA* transcripts.

Next, the effect of AUF1 silencing on the expression of the SNCA protein was evaluated. Western blotting of total protein extracts from SK-N-SH and HEK293 cells after AUF1 silencing for 48 h revealed a 33% (*p* < 0.001) and 20% (*p* < 0.01) decrease in total SNCA protein levels, respectively (figure 4*d*). Further, AUF1 depletion did not significantly affect *SNCA* transcript association with the ribosomes (figure 4*e*). This outcome was unpredicted, considering that AUF1 depletion increased half-life and steady-state levels of *SNCA* transcripts.

### 2.5. AUF1 regulates the maturation and nuclear export of *SNCA* transcripts

**Figure 5.**
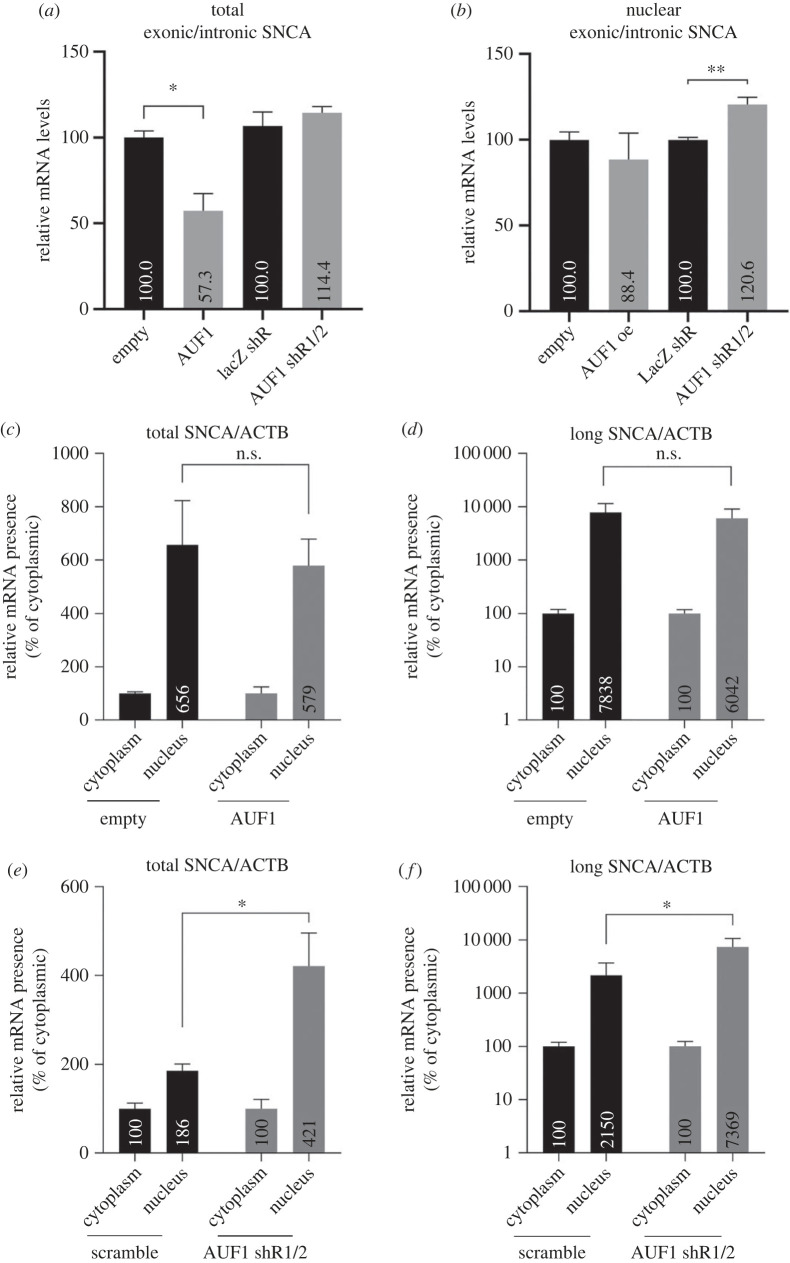
AUF1 attenuates *SNCA* pre-mRNA maturation and is indispensable for the nuclear export of *SNCA* mRNA. (*a,b*) RT-qPCR was used to measure the ratio of mature *SNCA* mRNA to pre-mRNA in nuclear and total extracts of SK-N-SH cells 48 h after AUF1 overexpression or silencing. AUF1 overexpression decreased the levels of mature *SNCA* mRNA in total lysates due to mRNA deadenylation, whereas AUF1 silencing significantly stimulated the maturation of *SNCA* pre-mRNA in the nucleus. (*c–f*) RT-qPCR was used to measure the expression of *SNCA* transcripts in nuclear and cytosolic extracts of SK-N-SH cells overexpressing or silencing AUF1. AUF1 overexpression did not significantly induce the nuclear export of total and long 3′UTR *SNCA* transcripts. AUF1 depletion blocked the nuclear export of *SNCA* transcripts. Data show the mean ± s.d. from at least 3 biological replicates (*p < 0.05, **p < 0.01).

Given that AUF1 shuttles between the nucleus and cytosol, it was hypothesized that the decline in SNCA protein expression following AUF1 depletion is because AUF1 is essential for either *SNCA* pre-mRNA maturation, as previously suggested for other targets [14], or mRNA export from the nucleus. To evaluate the above hypothesis, nuclear/cytosolic fractionation was conducted in AUF1 overexpressing or depleted SK-N-SH cells, followed by RT-qPCR to monitor intron-exon and inter-exonic *SNCA* mRNA levels in the nuclear and total cell extracts. AUF1 overexpression decreased the exon-to-intron ratio of *SNCA* transcripts in total cell lysates but not in nuclear extracts, as predicted for its role in *SNCA* mRNA cytoplasmic deadenylation (figure 5*a*), and did not significantly affect the maturation of *SNCA* pre-mRNAs (figure 5*b*). By contrast, AUF1 silencing increased by 21% (*p* < 0.01) mature (exonic) *SNCA* mRNA levels in the nucleus (figure 5*b*), indicating that AUF1 interferes with *SNCA* transcripts maturation. Further, whereas AUF1 overexpression marginally increased the nuclear export of total (13%, *p* = 0.95) and long (29%, *p* = 0.92) 3′UTR *SNCA* transcripts (figure 5*c,d*), AUF1 depletion decreased their nuclear export by 130% (*p* < 0.05) and 250% (*p* < 0.05), respectively (figure 5*e,f*). Collectively, AUF1 depletion promotes *SNCA* pre-mRNA maturation but impairs the nuclear export of mature transcripts, thereby impacting mRNA translation and total protein levels.

## 3. Discussion

Aberrant SNCA accumulation is a leading factor in initiating and aggravating neurodegeneration in sporadic and familial PD, DLB, and MSA. Delineating the mechanisms of SNCA regulation of expression is essential to any therapeutic intervention. Research has focused on understanding how defects in select pathways or catabolic processes (proteasome/autophagy) lead to SNCA protein misexpression. Little attention has been given to upstream events that regulate *SNCA* mRNA translation. Previously, we reported that the *SNCA* 5′UTR regulates protein output and harbours an internal ribosome entry site (IRES) element that permits protein synthesis during stress when most mRNAs are sequestered from translation [25]. Additionally, several studies have reported SNPs in the *SNCA* 3′UTR linked to PD [16,26–28] and changes in *SNCA* alternative polyadenylation in patients with PD [16,29], highlighting the contribution of *SNCA* 3′UTR regulatory elements in SNCA protein expression.

**Figure 6.**
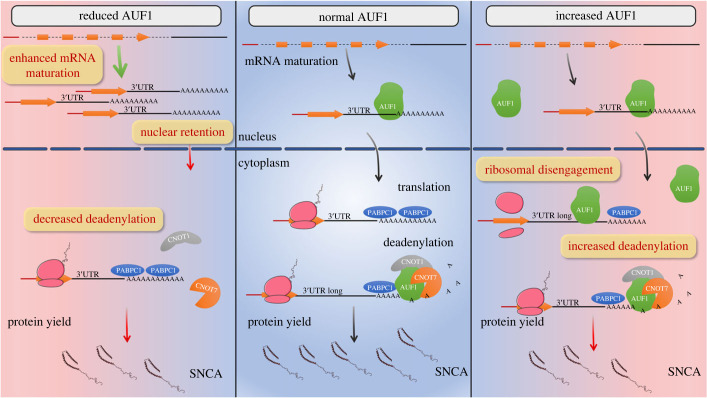
The regulation of SNCA expression by AUF1. In the nucleus, AUF1 interferes with *SNCA* pre-mRNA maturation and is essential for the nuclear export of mature transcripts. In the cytoplasm, AUF1 recruits the CNOT1-CNOT7 complex, deadenylating and destabilizing *SNCA* transcripts with shorter 3′UTR. Moreover, AUF1 inhibits the association of *SNCA* transcripts with the ribosomes, attenuating SNCA protein expression.

RBPs handle nearly all aspects of mRNA regulation of expression. To identify RBPs that bind *SNCA* mRNA, Marchese *et al*. first reported an *in vitro* protein library screening, identifying 27 top interactors. They prioritized focus on two RBPs, ELAVL1 and TIAR, and showed that TIAR, in particular, positively regulated *SNCA* mRNA translation [30]. Here, we, too, identified RBPs that bind *SNCA* 3′UTR, using murine brain lysates as a starting material. The protein lists from these two studies only overlapped on three proteins, Sub1/TCP4, HNRNPA1/ROA1, and ELAVL1/ELAV1, reflecting the different experimental approaches used. Subsequently, we chose to investigate the role of AUF1 in *SNCA* mRNA and protein expression. We demonstrated that AUF1 interferes with *SNCA* pre-mRNA maturation and is indispensable for the nuclear export of mature transcripts. AUF1 expression in the cytoplasm recruits the CNOT1-CNOT7 complex, deadenylating and destabilizing *SNCA* transcripts with shorter 3′UTR. In addition, AUF1 inhibits the association of all *SNCA* transcripts with the ribosomes, thereby attenuating SNCA protein expression (figure 6). These data suggest that, under physiological conditions, the levels and subcellular localization of AUF1 maintain optimum expression of SNCA and that conditions that alter AUF1 binding or distribution will profoundly affect *SNCA* expression: too little nuclear AUF1 will diminish *SNCA* mRNA nucleocytoplasmic shuttling, too much cytosolic AUF1 will destabilize mRNA and block translation. Coincidentally, the hypoglycemic agent metformin that induces nuclear retention of AUF1 disrupting its interaction with target mRNAs [31], is neuroprotective in different PD models in part by decreasing SNCA levels [32], phosphorylation [33,34] and aggregation [35]. Further, given that ELAVL1 binds *SNCA* 3′UTR [30] and AUF1 has been shown to compete with HuR for binding to specific U/AU-rich motifs in other mRNA targets [36,37], it is possible that the equilibrium between their levels may also affect SNCA expression.

In conclusion, this study identified RBPs that bound *SNCA* 3′UTR and detailed the complex interaction outcomes between AUF1 and *SNCA* mRNA. Subsequent studies will uncover the full spectrum of RBP interventions on *SNCA* mRNA expression, potentially revealing alternative paths to modulate SNCA levels for therapy.

## 4. Methods

### 4.1. Antibodies

The rabbit polyclonal anti-AUF1 antibody (07-260, Merck-Millipore, Darmstadt, Germany) was used in a dilution 1 : 2000. The mouse monoclonal antibody against SNCA (sc-7011) was purchased from Santa Cruz Biotechnology (Santa Cruz, CA, USA) and used in 1 : 1000 dilution. The rabbit polyclonal anti-CNOT1, anti-CNOT7, and anti-PABP1 antibodies were obtained from Proteintech (14 276-1-AP, 14 102-1-AP and 10 970-1-AP respectively, Proteintech Europe, Manchester, UK) and the mouse monoclonal anti-CNOT6 antibody (sc-81 231) was purchased from Santa Cruz Biotechnology (Santa Cruz, CA, USA). All four antibodies were used in a 1 : 1000 dilution. The mouse anti-GAPDH-HRP conjugated (HRP-60 004) was from Proteintech Europe (Manchester, UK) and was used in a 1 : 5000 dilution. The normal rabbit IgG (sc-2027) was from Santa Cruz Biotechnology (Santa Cruz, CA, USA). The mouse (#7076) and rabbit (#7074) HRP-conjugated secondary antibodies were from Cell Signaling Technologies (Danvers, MA, USA). The anti-puromycin antibody was purchased from Merck Millipore (MABE343, Darmstadt, Germany) and was used in a 1 : 3000 dilution.

### 4.2. Generation of DNA constructs

All primers used in this study are shown in electronic supplementary material, table S1.

The human AUF1 CDS (encoding the 42 kDa protein) was amplified by PCR using the proofreading Phusion polymerase (ThermoFisher) from cDNA prepared from total RNA extracted from SK-N-SH cells. The AUF1 PCR product was cloned between the KpnI and NotI restriction sites of the pENTR-GD plasmid, a kind gift of Dr A. Klinakis (BRFAA). Two AUF1 shRNA plasmids, targeting all *AUF1* transcripts*,* were prepared using the Block-iT U6 RNAi Entry vector Kit (ThermoFisher). Sanger sequencing verified the DNA sequence of all constructs at CeMIA SA (Larisa, Greece).

An alternative shRNA plasmid targeting all *AUF1* transcripts was kindly provided by Dr M. Gorospe and was used as an alternative to our shRNAs [38]; there was no difference in results. CNOT1 plasmid was kindly provided by Dr G. Stoecklin [21].

### 4.3. Peptide generation and 1-D nanoLC-MS/MS analysis

The extraction of proteins and the generation of peptides were performed as previously described [39]. In brief, the samples were treated in a water bath for 30 min, under mild sonication, with 7 M urea buffer and 80 mM triethyl ammonium bicarbonate (TEAB). The steps for the reduction and the alkylation of proteins were performed using dithiothreitol and iodoacetamide solutions at 10 mM and 55 mM, respectively. The final processing step included the tryptic digestion of extracted proteins for peptide generation.

### 4.4. LC-MS/MS analysis

As previously described, digested samples were analyzed using an LTQ Orbitrap Elite coupled to a Dionex 3000 HPLC system (Thermo Scientific, Rockford, IL, USA) [39]. Briefly, LC separation of peptides took place at a flow rate of 3 nl min^−1^ on two Thermo Scientific columns (PepMap RSLC, C18, 100 Å, 3 μm-bead-packed 15 cm column, and 2 μm-bead-packed 50 cm column). The mobile phases A and B were 0.1% formic acid in water and 99% acetonitrile in water. The gradient elution profile was as follows: 2.0% B (98.0% A) for 10 min, 2.0–35.0% B (98.0–65.0% A) for 325 min, 80.0% B (20.0% A) for 10 min, 2.0% B (98.0% A) for 10 min. Data were collected in the data-dependent MS/MS mode using a standard top-20 method. Full scan data were acquired at a resolving power of 60 000, with a maximum integration time of 250 msec. Scan range was fixed at 250 to 1250 *m/z*, and peptide fragmentation was performed in a higher energy collision dissociation (HCD) mode with a normalized collision energy of 36%.

MS/MS spectra were obtained with 15 000 resolving power and a maximum integration time of 120 ms. The measurements were made using *m/z* 445.120025 as lock mass. Dynamic exclusion settings were set to repeat count 1, repeat duration 30 s, exclusion duration 120 s, and exclusion mass width 0.6*m/z* (low) and 1.6*m/z* (high).

The SEQUEST algorithm in the Proteome Discoverer was used to process raw data files. MS/MS searches were performed using a 20 ppm parent ion mass tolerance and a 0.05 fragment mass tolerance. Trypsin was selected as the cleavage enzyme with up to 2 missed cleavage points. Cysteine methylthio modification was selected as a fixed modification, and methionine oxidation was selected as a variable. Peptide identifications were valid at 1% False Discovery Rate (*q*-value 0.01) (percolator maximum Delta Cn was 0.05). The minimum length of acceptable identified peptides was set as 6 amino acids.

### 4.5. Cell culture and transfection

Human SK-N-SH and HEK293A cells were grown in high-glucose DMEM (Sigma-Aldrich, St. Louis, MO, USA) supplemented with 10% fetal bovine serum (FBS) (ThermoFisher, Waltham, MA, USA) and 1% penicillin/streptomycin (Sigma-Aldrich). Cells were maintained at 37°C in a humidified 5% CO_2_ incubator (ThermoForma, ThermoFisher). Cells were transfected at plating with plasmids using the jetOPTIMUS reagent according to manufacturer instructions (Polyplus, Illkirch, France). EmGFP plasmid transfection indicated that efficiency was approximately 80% for both cell lines at 48 h. Cells were harvested 48 h after transfection.

### 4.6. Affinity pull-down of biotinylated RNA for protein-RNA complex detection

The different domains of the *SNCA* mRNA (5′UTR, CDS, 3′UTR_570_, and 3′UTR_2529_) were amplified by PCR from SK-N-SH cDNA using specific primers. In all cases, the forward primer included the T7 RNA polymerase promoter sequence 5′-AGTAATACACTCACTATAGGG-3′, which is required for transcription.

For the *in vitro* transcription, a 20 µl reaction containing 0.75 µg PCR product, 1 × T7 buffer, 2 µl 50 mM DTT, 0.5 µl RNAse inhibitors (RNAaseOUT, ThermoFisher), 2 µl (20 mM A/U/G, 16.3 mM C, 3.7 mM biotin-11-CTP, Roche) NTPs, 1 µl T7 polymerase (Takara Bio, Kusatsu, Japan) was incubated at 42°C for 2 h. The template DNA was removed by RNase-free DNAase I (NEB) treatment for 20 min at 37°C, and the transcribed RNA was isolated by LiCl-mediated precipitation. The RNA probe was prepared by adding 2 *μ*g of RNA to 100 µl structure buffer comprised of 10 mM Tris pH7.0, 0.1 M KCl, and 10 mM MgCl_2_. It was then heated to 90°C for 2 min, placed on ice for 2 min, and left to fold at room temperature for 20–30 min.

A single postnatal day 3 mouse brain or a 10 cm plate of SK-N-SH cells (approx. 10^7^ cells) was required per pull-down condition. SK-N-SH cells were initially washed with PBS before resuspending in 350 µl of ice-cold NT2 buffer comprised of 50 mM Tris-HCl pH7.4, 150 mM NaCl, 1 mM MgCl_2_, 0.05% NP-40, and supplemented with 1× cOmplete Protease Inhibitor Cocktail (Roche, Basel, Switzerland). Lysates were vortexed briefly and placed on a rotating mixer at 4°C for 45 min, after which they were centrifuged at 16 000 × *g* for 10 min at 4°C to collect the supernatant.

For the RNA-protein pull-down, the cell lysate and the RNA probe were mixed and incubated at RT for 2 h on a rotating mixer. Then, 50 µl streptavidin magnetic beads, previously washed twice with NT2 buffer, were added, and the slurry was further incubated at RT for 2 h on a rotating mixer. Finally, the beads were washed 3 times with 250 µl NT2 buffer to clear non-specific protein binding, incubated with 100 µl NT2 supplemented with 1 × Laemmli buffer at RT for 15 min, and boiled for 10–15 min to release protein pull-downs.

### 4.7. Immunoprecipitation of RNP complexes (RIP)

To identify RNAs bound to AUF1, protein A/G Sepharose beads (Santa Cruz Biotechnology) were coated overnight at 4°C with 2 µg anti-AUF1 or anti-IgG antibody in NT2 buffer containing 5% BSA, under constant agitation. The following day, the beads were washed three times with NT2 buffer. SK-N-SH extracts were prepared by incubating for 30 min 10^7^ cells with ice-cold PLB lysis buffer comprised of 10 mM HEPES pH7.0, 100 mM KCl, 5 mM MgCl_2_, 0.5% NP-40, 1 mM DTT, and supplemented with 1 × cOmplete Protease Inhibitor Cocktail and 40 U/mL RNAseOUT before pelleting the debris with cold centrifugation at 16 000 × g for 10 min. The cell extracts were combined with the antibody-coated beads and placed on a rotating mixer at 4°C for 4 h, excluding a small quantity set aside as input. The beads were washed 3 times with NT2 to remove non-specific protein/RNA binding. They were then treated with proteinase K to digest proteins bound to the beads before adding Tri Reagent (Molecular Research Centre Inc, Cincinnati, OH, USA) for RNA extraction.

### 4.8. Total RNA extraction, cDNA synthesis and PCR

Total RNA was extracted from SK-N-SH cells using the TRI Reagent according to the manufacturer's instructions. RNA amount and purity were assessed by measuring the absorbance at 260 and 280 nm. The A260/280 ratio of all samples was greater than 1.9. First-strand cDNA was synthesized using 0.5 µg total RNA and random hexamers according to the M-MLV reverse transcriptase protocol (ThermoFisher). For miRNA detection, a polyadenylation step was included before reverse transcription using poly(A) polymerase (NEB) as previously described [40]. The resulting cDNA was diluted 11 times with nuclease-free water and stored at −80°C until use in quantitative PCR (qPCR). The qPCR assay used the Kapa SYBR Fast Universal 2 × qPCR Master Mix (Kapa Biosystems, Roche, Basel, Switzerland) and was carried out in 96-well PCR microplates (Azenta, Burlington, MA, USA) on the CFX OPUS real-time PCR system (BioRad, Richmond, California, USA). Each qPCR run included reverse-transcription-negative controls, and each sample was tested in triplicate. Data were analysed using the 2-ΔΔC_T_ method [40], with *GAPDH* serving as a normalization standard (for primers, see electronic supplementary material, table S1).

### 4.9. mRNA half-life measurement

Forty-eight hours after transfection with AUF1 or control plasmids, 5 µg ml^−1^ actinomycin D (MedChemExpress, Monmouth Junction, NJ, USA) was added to the cell culture medium, and SK-N-SH cells were harvested 0, 3, 6 and 9 h later with TRI Reagent. Total RNA was extracted and subjected to RT-qPCR analysis. The one-phase decay nonlinear regression analysis was conducted in GraphPad Prism (Y_0_ = 100).

### 4.10. Extended poly(A) test (ePAT) method and melting curve predictions

Poly(A) tail length was analyzed using the ePAT method with minor modifications [18]. For the initial assembly, 0.5 µg of total RNA and 1 µl of ePAT-anchor primer were combined in a final volume of 8 µl, incubated at 80°C for 5 min, and cooled to RT. Then, 12 µl containing 4 µl dH_2_O, 4 µl 5 × First strand Buffer, 2 µl 100 mM DTT, 1 µl 10 mM dNTPs, 0.5 µl RNaseOUT and 0.5 µl Μ-MLV were added. The sample was incubated at 25°C for 10 min, 37°C for 1 h, and 75°C for 15 min. For the PCR reactions, cDNA was diluted 1 : 8 with ddH_2_O, and 10 µl was used as a template for each 20 µl reaction.

For melting curve predictions, the amplified sequences with different size poly(A) tails were uploaded to uMeltQUARTZ software [41].

### 4.11. Nucleocytosolic fractionation

SK-N-SH cells were harvested in ice-cold HLB buffer containing 10 mM Tris pH7.4, 10 mM NaCl, 3 mM MgCl_2_, 1 mM EGTA, 0.1% NP40, and supplemented with RNAseOUT, and incubated for 10 min on ice. Lysates were centrifuged at 800 × g for 3 min at 4°C to separate cytoplasmic (supernatant) and nuclear (pellet) fractions. The pellets were washed with HLB buffer three times before resuspending in TRI Reagent. During the phase separation step of RNA extraction, the nuclear fraction was incubated at 65°C for 10 min to release membrane-bound mRNAs.

### 4.12. RPL22 riboTag assay

RiboTag is used to detect changes in mRNA transcript engagement with the ribosome using RT-qPCR. Approximately 10^6^ cells (one 35 mM dish of cells) were required per condition. Forty-eight hours post-co-transfection with FLAG-tagged RPL22 and AUF1 plasmids, cells were washed twice with ice-cold PBS, harvested with 750 µl PLB lysis buffer, and incubated on ice for 30 min. The cell debris was pelleted by cold centrifugation at 16 000 × g for 10 min, and the supernatant was transferred to a new tube. 25 µl of anti-FLAG G1 resin (MedChemExpress, NJ, USA) was required per condition and was prepared by washing 3 times with 500 µl TBS before resuspending in 250 µl TBS containing 40 U ml^−1^ RNAseOUT. After reserving 10% of the supernatant as input, the remaining lysate was incubated with the beads for 4 h at 4°C on a rotating mixer. The beads were washed thrice with 500 µl NT2 buffer before resuspending in 75 µl NT2 buffer. TRI Reagent was added to the beads and inputs for RNA extraction.

### 4.13. Co-Immunoprecipitation

Forty-eight hours after 1.5 × 10^7^ SK-N-SH cells were co-transfected with CNOT1 and either empty- or AUF1-expressing plasmids, the cells were resuspended in 2 ml of ice-cold non-denaturing PLB lysis buffer containing 10 mM HEPES pH 7.0, 100 mM KCl, 5 mM MgCl_2_, 0.5% NP-40, and 1 × cOmplete Protease Inhibitor Cocktail. After 30 min on a rotating mixer at 4°C, the cell suspension was centrifuged at 16 000 × g for 15 min at 4°C, and the supernatant was transferred to new tubes. Then, the supernatant was divided into two tubes containing 2 µg of either anti-goat IgG or anti-CNOT1 antibody and incubated overnight at 4°C on a rotating mixer.

PureProteome Protein A/G mix magnetic bead suspension (Merck/Millipore, Burlington, MA, USA) was washed thrice with PBS containing 0.1% Tween-20 and blocked for an hour at RT with PBS containing 2% BSA and 0.1% Tween-20. The bead slurry was then washed thrice with PBS and equilibrated with NT2 washing/elution buffer containing 50 mM Tris-HCl pH 7.4, 250 mM NaCl, 1 mM MgCl_2_, and 0.05% Tween-20. Each protein lysate was incubated with 30 µl of bead slurry in microcentrifuge tubes for 30 min at RT on a rotating mixer. The beads were subsequently washed three times with NT2 buffer. Immunoprecipitated proteins were eluted by resuspending each bead slurry in 100 µl of NT2 buffer supplemented with 6 × Laemmli buffer containing 250 mM *Τ*ris-HCl pH6.8, 6% SDS, 30% β-mercaptoethanol, 40% glycerol, and 0.005% bromophenol blue and incubating for 10 min at 98°C, followed by brief centrifugation.

### 4.14. Preparation of whole protein extracts and Western blotting

Whole-cell lysates were harvested using an ice-cold RIPA lysis buffer comprised of 25 mM Tris pH7.5, 150 mM NaCl, 1.5 mM EDTA, 1% Triton X-100, 0.16% sodium deoxycholate, 0.16% SDS and 1 × cOmplete Protease Inhibitor Cocktail. After incubation for 30 min on ice, cell suspensions were centrifuged for 30 min at 16 000 × g at 4°C. The supernatants were afterward transferred to new tubes and stored at −80°C before use. Total protein amounts were quantified using the Bradford assay according to the manufacturer's instructions (BioRad).

For immunoblotting, equal amounts of protein extracts were supplemented with 6 × SDS sample buffer containing 375 mM Tris pH6.8, 10% SDS, 50% glycerol, 10% β-mercaptoethanol, 0.03% bromophenol blue, heated for 5 min at 100°C, separated by 12% or 15% SDS-PAGE under reducing conditions and transferred to a Protran nitrocellulose membrane (Amersham/Merck, St. Louis, MO, USA). Nitrocellulose membranes were then blocked with Tris-buffered saline (TBS) containing 5% non-fat milk and 0.1% Tween-20 (TBS-T) for 1 h at RT and afterward were probed with the respective primary antibodies. All primary antibodies were diluted in TBS-T and incubated overnight at 4°C. All secondary HRP-conjugated antibodies were diluted in TBS-T but incubated for 1 h at RT. The immunoreactive bands were visualized with the enhanced chemiluminescence (ECL) method using the Clarity or Clarity Max ECL reagents (BioRad Laboratories, Richmond, CA, USA) according to the manufacturer's instructions. Densitometric analysis of images was performed using the image analysis software ImageJ (NIH, USA). GAPDH was used for normalization.

### 4.15. Surface sensing of translation (SUnSET) method

This technique is used to detect changes in protein synthesis rates in whole-cell lysates using Western blotting [24]. It uses an anti-puromycin antibody for the immunological detection of puromycin-labelled peptides. Specifically, 48 h post-transfection, 1 µΜ puromycin (P8833, Sigma-Aldrich, Canada) was added to the cell culture medium, and SK-N-SH cells were harvested 30 min later in ice-cold RIPA buffer. Protein extracts were supplemented with 6 × SDS sample buffer and separated by 10% SDS-PAGE. Blocked membranes were incubated overnight at 4°C with anti-puromycin antibody in TBS-T, then at RT for 1 h with anti-mouse HRP secondary antibody and developed with ECL.

### 4.16. Gene ontology analysis

Gene Ontology (GO) ‘Molecular function’ and ‘Biological process’ analyses were performed using the WebGestalt 2019 gene set analysis toolkit with an FDR < 0.05 [42]. The *Homo sapiens* genome protein-coding database was used as a reference.

### 4.17. Statistical analysis

The data were compiled and analysed from at least three independent biological replicates and are presented as the mean ± standard deviation (SD). For paired samples, the statistical significance of differences between two groups was determined by two-tailed Student's *t*-test. Multiple comparisons were performed using one-way ANOVA, followed by Dunnett's post hoc analysis for repeated measurements. *p*-values < 0.05 were set as the cutoff for statistically significant differences. GraphPad Prism software was used for statistical analysis (version 8.0.0; San Diego, California, USA).

## Data Availability

All data generated during this study are included in this published article and its electronic supplementary material [43].
